# Post Kidney Transplant Cyclosporine-Induced Acute Pancreatitis

**DOI:** 10.7759/cureus.24519

**Published:** 2022-04-27

**Authors:** Enas Al-Najada, Asem Alobaidat, Mo'ath M Rabab'ah, Moh'd Bani Salameh, Lean Alkhatib

**Affiliations:** 1 Nephrology, Royal Medical Services, Amman, JOR; 2 Pharmacology and Therapeutics, Royal Medical Services, Amman, JOR; 3 Internal Medicine, Royal Medical Services, Amman, JOR

**Keywords:** acute pancreatitis, post transplantation, transplant nephrology, medication induced pancreatitis, cyclosporine-a

## Abstract

Drug-induced pancreatitis (DIP) is a rare cause of acute pancreatitis. Efforts have been made to assess the relationship between many drugs and acute pancreatitis. Also, studies have been held to investigate the possible mechanisms of DIP. Cyclosporine is one of the immunosuppressive agents that is still under investigation regarding its association with acute pancreatitis.

We report a case of a 21-year-old male patient post kidney transplant who presented with a picture of acute pancreatitis; upon further investigation, the diagnosis of cyclosporine-induced pancreatitis was made by ruling out all other possible causes of acute pancreatitis. Furthermore, he showed significant improvement and was discharged home upon stopping cyclosporine and replacing it with sirolimus, and there was no relapse of pancreatitis in three months of follow-up.

Our case provides evidence that cyclosporine can be a possible cause of pancreatitis in post kidney transplant patients receiving cyclosporine, and how early detection of cyclosporine-induced pancreatitis can significantly improve the patient's condition.

## Introduction

Acute pancreatitis is an uncommon potentially fatal condition in post kidney transplant patients [1]. It can be attributed to several factors including immunosuppressive agents [2]. Drug-induced pancreatitis (DIP) is considered rare and accounts for 0.1-2% of acute pancreatitis cases [3]. The exact mechanism of DIP is still under investigation, but possible mechanisms were suggested by other studies. The diagnosis of DIP is made by developing symptoms while being on a doubtful drug and by eliminating all other possible differential diagnoses of acute pancreatitis. Re-challenge test to assess the association between a particular drug and pancreatitis is ethically limited. DIP is treated by supportive care, stopping the offending agent, and using another drug when required [3,4].

Cyclosporine is one of the immunosuppressive drugs used in post kidney transplants and it is known to cause nephrotoxicity, hypertension, dyslipidemia, and the patient becomes susceptible to infections [5,6]. However, cyclosporine-induced pancreatitis in those patients was rarely reported previously. Here, we present a rare case of cyclosporine-induced pancreatitis in a 21-year-old post kidney transplant male recipient who was receiving cyclosporine after transplantation.

## Case presentation

A 21-year-old male received a renal transplant from his living mother. Post kidney transplant, he was maintained on a triple immunosuppressive therapy that consisted of cyclosporine 150 mg twice daily, mycophenolate mofetil 1000 mg twice daily, and prednisolone 5 mg once daily with a regular follow-up. The surgery was successful and his post-op course was smooth without any complications.

On day +93, after transplantation, he presented to our emergency department with severe epigastric pain radiating to his back, nausea, vomiting, and loss of appetite for three days prior to admission. On physical examination, vital signs showed a body temperature of 37°C, a blood pressure of 100/60 mmHg, and tachycardia (heart rate of 120 beats per minute). In addition, he had moderate epigastric tenderness. Initial laboratory tests revealed a significant elevation of serum amylase level (1122 IU/L), slightly elevated white blood cell count (WBC) (11.4 x 10^3^/uL), and high c-reactive protein (CRP) (17 mg/dL). His alanine aminotransferase (ALT) (5.8 IU/L), aspartate aminotransferase (AST) (10.3 IU/L), total bilirubin (0.954 mg/dL), direct bilirubin (0.23 mg/dL), and alkaline phosphatase were normal. His creatinine (1.1 mg/dL) was normal but his blood urea nitrogen (BUN) (30 mg/dL) was high. His serum calcium was normal (8.98 mg/dL). His serum triglyceride level was normal (143 mg/dL). His cyclosporine C2 level was low (1000 ng/mL) (Table 1).

**Table 1:** Basic laboratory investigations. WBC: white blood cell count; AST: aspartate aminotransferase; BUN: blood urea nitrogen; CRP: c-reactive protein; ALT: alanine aminotransferase; ALP: alkaline phosphatase

Laboratory	Result	Reference range
Amylase	1122 IU/L	40-140 IU/L
WBC	11.4 × 10^3^/uL	4.5-5.5 × 10^3^/uL
CRP	17 mg/dL	0.8-1.0 mg/dL
ALT	5.8 U/L	0-41 U/L
AST	10.3 U/L	0-37 U/L
Total bilirubin	0.954 mg/dL	0.1-1.2 mg/dL
Direct bilirubin	0.23 mg/dL	0.1-0.3 mg/dL
ALP	101 U/L	40-129 U/L
Creatinine	1.1 mg/dL	0.5-1.2 mg/dL
BUN	30 mg/dL	6-20 mg/dL
Calcium	8.98 mg/dL	8.4-10.5 mg/dL
Triglycerides	143 mg/dL	50-200 mg/dL
Cyclosporine C2	1000 ng/mL	4-6 months post-transplant 1100 ng/mL

**Figure 1:**
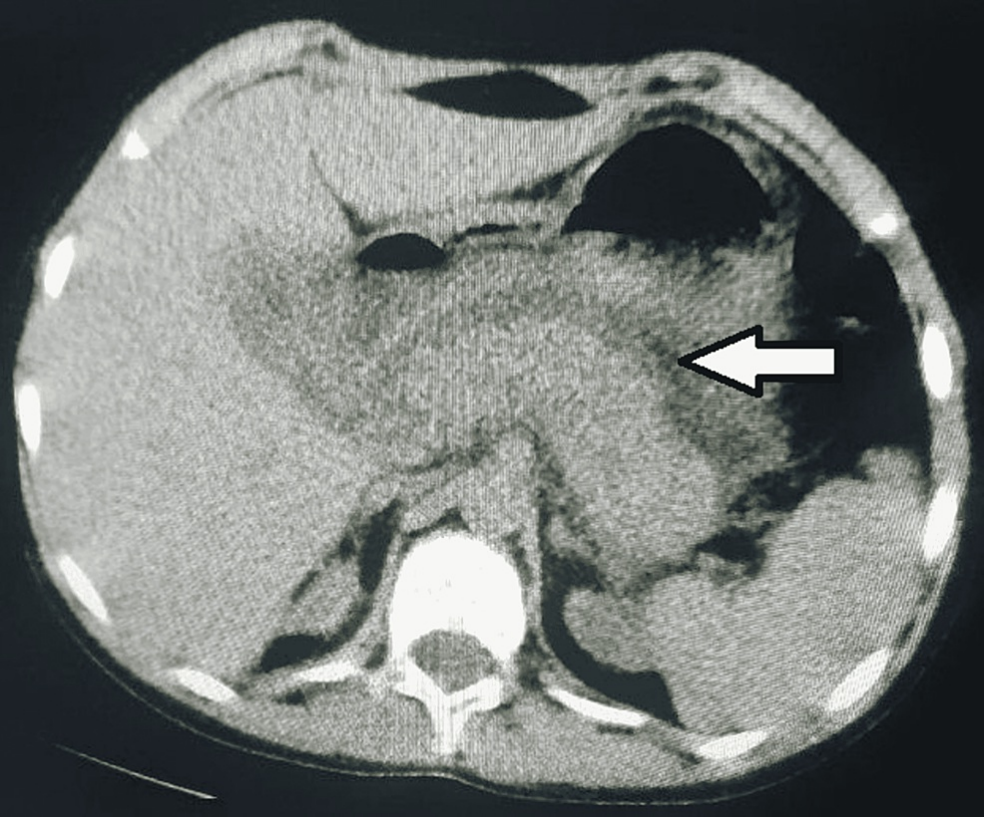
Abdominal CT demonstrating diffuse pancreatic enlargement with peripancreatic fluid and fat stranding (white arrow).

Serological tests for viral hepatitis and cytomegalovirus (CMV) were negative. Also, his CMV polymerase chain reaction (PCR) test was negative. His computed tomography (CT) scan showed diffuse pancreatic parenchymal enlargement with peripancreatic fluid and surrounding fat stranding. Those findings are consistent with interstitial edematous pancreatitis (Figure 1).

Based on the clinical presentation, laboratory test results, and his CT findings, the diagnosis of acute pancreatitis was established. Drug-induced pancreatitis was suggested after ruling out hypercalcemia, hypertriglyceridemia, viral infections, and biliary tract obstruction. Prednisolone was stopped and he was managed by fasting, intravenous fluids, and analgesia with no improvement in his condition after one week of conservative management, then we stopped mycophenolate mofetil with no improvement even after 10 days of conservative management. After that, we stopped cyclosporine and the patient started improving and was discharged home two weeks following the withdrawal of cyclosporine. Cyclosporine was replaced by sirolimus 2 mg once daily and his mycophenolate mofetil and prednisolone were resumed. There was no relapse in three months of follow-up with a stable graft function.

## Discussion

Acute pancreatitis is an uncommon potentially fatal condition in post kidney transplant patients [1]. It can be attributed to several factors including hypercalcemia, dyslipidemia, hyperparathyroidism, biliary tract obstruction, viral infections such as cytomegalovirus (CMV), and immunosuppressive agents [1,7]. The mechanism of drug-induced pancreatitis (DIP) is unclear. Possible mechanisms are direct toxicity, drug-induced hypertriglyceridemia, and allergic reactions [3]. DIP is managed by stopping the offending drug and starting another agent when required [4]. A classification system for DIP was established by Badalov et al. [8]. It consists of four categories that indicate the association between a particular drug and pancreatitis based on evidence. Among the immunosuppressive agents used in post kidney transplant, azathioprine is a class Ib drug, prednisolone and cyclosporine are class III drugs, and tacrolimus is a class IV drug (Table 2) [4,5,8].

**Table 2:** Badalov classification of drug-induced pancreatitis.

Category	Criteria
Class Ia	At least 1 case report with positive rechallenge, excluding all other causes, such as alcohol, hypertriglyceridemia, gallstones, and other drugs
Class Ib	At least 1 case report with positive rechallenge; however, other causes, such as alcohol, hypertriglyceridemia, gallstones, and other drugs were not ruled out
Class II	At least 4 cases in the literature; consistent latency (75% of cases)
Class III	At least 2 cases in the literature; no consistent latency among cases -No rechallenge
Class IV	Drugs not fitting in the earlier-described classes, single case report published in medical literature, without rechallenge

Cyclosporine has many side effects including nephrotoxicity, hypertension, dyslipidemia, and infections [6]. However, cyclosporine-induced pancreatitis post kidney transplant was rarely reported in the literature. A study held in 1988 by the National Kidney Foundation showed that four patients out of 105 post kidney transplant cyclosporine-managed patients developed acute pancreatitis. Those patients had high, medium, and low cyclosporine trough levels at the time of diagnosis. Cyclosporine was strongly suggested as the patients showed improvement upon reducing its dose or being discontinued and replaced by another agent. Also, the microscopic exam showed no steroid-related changes [9]. Another study held in 1998 on rats showed that high doses of cyclosporine exacerbate post-transplant pancreatitis, while low doses do not [10].

In our case, the diagnosis of cyclosporine-induced pancreatitis was made by meeting the first three criteria of Mallory and Kern classification for DIP - (1) developing pancreatitis while being treated with a drug (2) excluding other causes of pancreatitis, (3) recovery upon discontinuation of the drug, and (4) relapse of pancreatitis after reinitiation of the drug [11]. For that reason, cyclosporine was identified as the cause of pancreatitis in our case.

## Conclusions

In our case, the diagnosis of cyclosporine-induced pancreatitis was made by ruling out more common causes of acute pancreatitis. Moreover, it was strongly suggested by the improvement in our patient showed upon stopping cyclosporine and replacing it with sirolimus. This study demonstrates that cyclosporine can be a possible cause of acute pancreatitis in post kidney transplant patients on cyclosporine treatment. However, further studies are warranted to study pancreatitis as a side effect of cyclosporine.
